# Anti-Cancer Effects and Tumor Marker Role of Glutathione *S*-Transferase Mu 5 in Human Bladder Cancer

**DOI:** 10.3390/ijms22063056

**Published:** 2021-03-17

**Authors:** Yeong-Chin Jou, Shou-Chieh Wang, Yuan-Chang Dia, Shou-Tsung Wang, Min-Hua Yu, Hsin-Yi Yang, Lei-Chin Chen, Cheng-Huang Shen, Yi-Wen Liu

**Affiliations:** 1 Department of Urology, Ditmanson Medical Foundation Chiayi Christian Hospital, Chiayi 600, Taiwan; 01729@cych.org.tw; 2 Department of Health and Nutrition Biotechnology, Asian University, Taichung 41354, Taiwan; 3 Department of Microbiology, Immunology and Biopharmaceuticals, College of Life Sciences, National Chiayi University, Chiayi 600, Taiwan; johnsjwang@gmail.com (S.-C.W.); cych03884@gmail.com (Y.-C.D.); s1020481@alumni.ncyu.edu.tw (S.-T.W.); minayu86@gmail.com (M.-H.Y.); 4 Department of Food Science, College of Life Sciences, National Chiayi University, Chiayi 600, Taiwan; 5 Division of Nephrology, Department of Internal Medicine, Kuang Tien General Hospital, Taichung 437, Taiwan; 6 Department of Pathology, Ditmanson Medical Foundation Chiayi Christian Hospital, Chiayi 600, Taiwan; 7 Department of Clinical Medicine, Ditmanson Medical Foundation Chiayi Christian Hospital, Chiayi 600, Taiwan; cych13018@gmail.com; 8 Department of Nutrition, I-Shou University, Kaohsiung 82445, Taiwan; lcchen@isu.edu.tw

**Keywords:** bladder cancer, DNA methylation, glutathione *S*-transferase Mu, glutathione, tumor suppressor

## Abstract

Our previous study demonstrated that the glutathione *S*-transferase Mu 5 (GSTM5) gene is highly CpG-methylated in bladder cancer cells and that demethylation by 5-aza-dC activates GSTM5 gene expression. The aim of the present study was to investigate the role of GSTM5 in bladder cancer. The levels of GSTM5 gene expression and DNA methylation were analyzed in patients with bladder cancer, and functional studies of GSTM5 were conducted using GSTM5 overexpression in cultured bladder cancer cells. Clinical analysis revealed that the GSTM5 mRNA expression was lower in bladder cancer tissues than in normal tissues and that the level of GSTM5 DNA methylation was higher in bladder cancer tissues than in normal urine pellets. Overexpression of GSTM5 decreased cell proliferation, migration and colony formation capacity. Glutathione (GSH) assay results indicated that cellular GSH concentration was decreased by GSTM5 expression and that GSH supplementation reversed the decrease in proliferation and migration of cells overexpressing GSTM5. By contrast, a GSH synthesis inhibitor significantly decreased 5637 cell GSH levels, survival and migration. Furthermore, GSTM5 overexpression inhibited the adhesion of cells to the extracellular matrix protein fibronectin. To elucidate the effect of GSTM5 on anticancer drugs used to treat bladder cancer, cellular viability was compared between cells with or without GSTM5 overexpression. GSTM5-overexpressed cells showed no significant change in the cytotoxicity of cisplatin or mitomycin C in 5637, RT4 and BFTC 905 cells. Though a degree of resistance to doxorubicin was noted in 5637 cells overexpressing GSTM5, no such resistance was observed in RT4 and BFTC 905 cells. In summary, GSTM5 plays a tumor suppressor role in bladder cancer cells without significantly affecting chemoresistance to cisplatin and mitomycin C, and the cellular GSH levels highlight a key mechanism underlying the cancer inhibition effect of GSTM5. These findings suggest that low gene expression and high DNA methylation levels of GSTM5 may act as tumor markers for bladder cancer.

## 1. Introduction

Bladder cancer is the fourth most common type of cancer among men in the United States [1] and is the ninth most common cancer type worldwide [2]. Although bladder cancer is the 13th most lethal cancer type globally [2], its high recurrence impairs patient quality of life and results in considerable economic burden. Recently, immunotherapy has provided hope for the treatment of metastatic platinum-refractory bladder cancer but is only effective in patients with programmed cell death 1 ligand 1-positive status [3]. Therefore, it is important to prevent the development of bladder tumors by elucidating the factors involved in their carcinogenesis and progression. There are various risk factors for the development of bladder cancer, including exposure to arylamines [4], smoking [5], exposure to environmental arsenic [6,7], aging and hereditary influences. Among these factors, cigarette smoke is the single most prevalent cause of bladder cancer [8].

Gene polymorphism is also a factor for bladder cancer development. Glutathione *S*-transferases (GSTs), a family of phase 2 detoxification enzymes, play an important role in the metabolism and/or detoxification of various endo- and xenobiotics, including drugs [9]. GSTs catalyze the conjugation of glutathione (GSH) to a wide variety of xenobiotics. This detoxification ability plays an important role in cellular protection from the environment and oxidative stress but is also implicated in cellular resistance to various chemotherapy drugs [10]. GSTs can be categorized into different families according to similarities in amino acid sequence. GSTmu (GSTM) is one of the largest GST families, which contains five members, GSTM1-M5. It is known that approximately 50% of the population possess the GSTM1-null genotype, which results in a lack of GSTM1 enzyme activity [11]. Furthermore, loss of the GSTM1 gene is known to be correlated with human bladder cancer [11,12]. Smoking also increases the odds ratio of bladder cancer in individuals with the GSTM1-null genotype [12,13]. Cigarette smoke contains various carcinogens, including nitrosamines, and the bladder carcinogen *N*-butyl *N*-(4-hydroxybutyl) nitrosamine has been shown to induce mouse bladder carcinogenesis accompanied by decreasing GSTM1 gene expression [14]. The human genome database indicates that GSTM1-M5 is located on chromosome 1 and in close proximity to each other [15]. Furthermore, genes in the same GST family have a greater opportunity to play as substitutes for one another. For example, GSTM2 has been reported to functionally compensate for GSTM1-null in Indian people [16]. Moreover, a protective genotype with higher GSTM3 expression exhibits a lower breast cancer risk when GSTM1 is absent [17].

The role of GSTM2 has been studied in lung cancer, where it was found to decrease benzo[a]pyrene-induced DNA damage in lung cancer cells [18,19]. DNA methylation of the GSTM2 gene promoter decreases its gene expression by inhibiting Sp1 binding [20], and GSTM2 expression suppresses the migration of lung cancer cells [21]. A global analysis of promoter methylation in treatment-naïve urothelial cancer suggests that GSTM3 is one of the hypermethylated genes in invasive urothelial carcinoma [22]. In addition, other published reports have indicated an association between GSTM5 and cancers. A study of Barrett’s adenocarcinoma indicated that the gene expression of GSTM2, GSTM3 and GSTM5, but not GSTM4, was lower in tumors than in normal tissues and that DNA methylation also regulates gene expression [23]. In our previous study, the GSTM5 gene was found to be heavily CpG methylated and expressed at low levels in human bladder cancer cells. After treatment with the DNA methylation inhibitor 5-aza-2′-deoxycytidine (5-aza-dC), the methylation level of GSTM5 was decreased, resulting in an increase in GSTM5 gene expression in 5637 and J82 cells [24]. In the present study, the DNA methylation level of human GSTM5 was analyzed in patients with bladder cancer and normal subjects, and GSTM5 was overexpressed in bladder cancer cells to assess changes in cancer-associated characteristics.

## 2. Results

### 2.1. GSTM5 mRNA Expression Is Downregulated in Bladder Cancer Tissues

**Figure 1:**
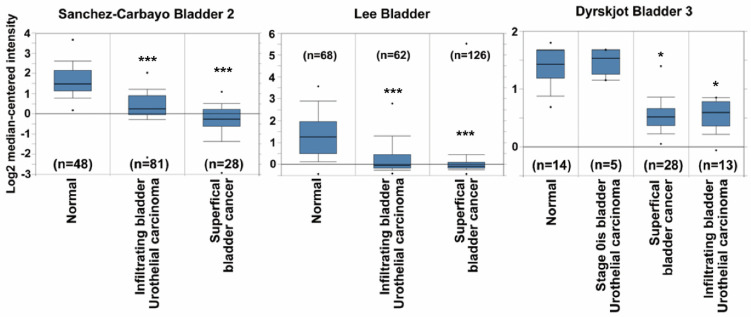
Glutathione *S*-transferase Mu 5 (GSTM5) mRNA expression is downregulated in patients with bladder cancer from the Oncomine database. Three mRNA databases exhibited different expression levels of GSTM5 mRNA between normal and cancer tissues (left panel: Sanchez-Carbayo bladder 2, middle panel: Lee bladder, right panel: Dyrskjot bladder 3). The dots are the maximum and minimum, the box contains 75% to 25% values, the line in box is median, and the lines outside the box are the values of 90% and 10%. The n means bladder sample number, * *p* < 1 × 10^−7^; *** *p* < 1 × 10^−9^ compared to normal.

The GSTM5 mRNA expression data of patients with bladder cancer were extracted from the publicly available Oncomine database. After data-mining for GSTM5 and bladder cancer in cancer vs. normal differential analysis (*p* < 0.001, fold change >1.5, gene rank the top 10%), three mRNA databases exhibited differential expression (Sanchez-Carbayo bladder 2, Lee bladder and Dyrskjot bladder 3). In these three databases, GSTM5 mRNA was downregulated in superficial and infiltrating bladder tumor tissues compared with normal tissues (Figure 1), suggesting that GSTM5 may play a tumor suppressor role in human bladder cancer.

### 2.2. DNA Methylation Level of the GSTM5 Gene Promoter Is Increased in Bladder Cancer Tissues

**Figure 2:**
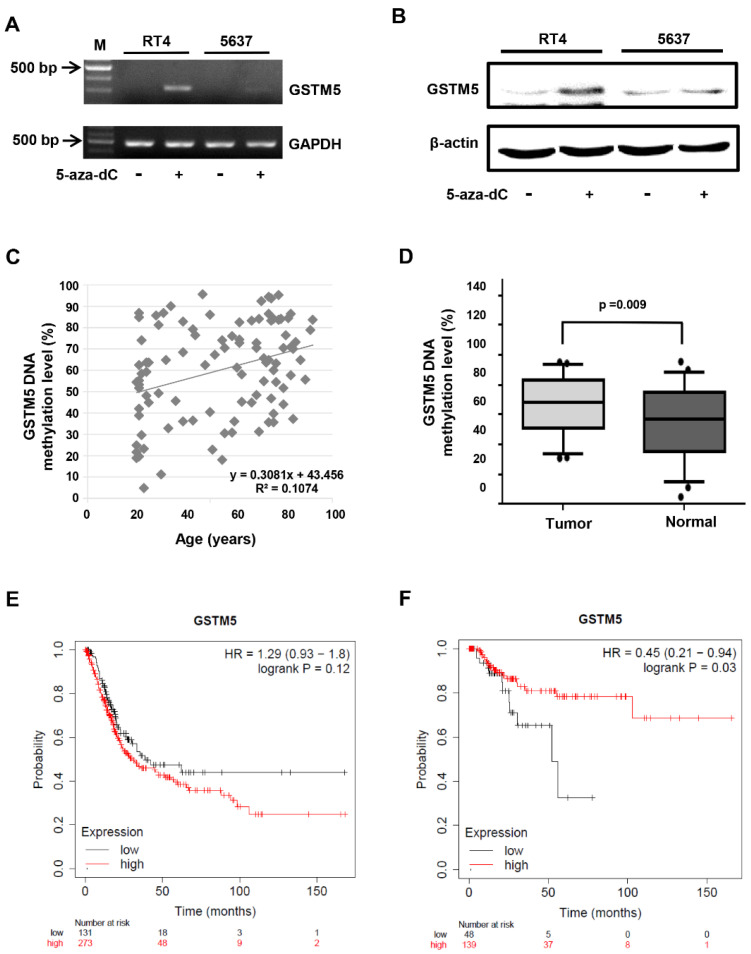
The status of GSTM5 DNA methylation in bladder cancer cells and clinical samples. (**A**) The effect of 10 µM 5-aza-dC on GSTM5 mRNA expression. (**B**) The effect of 10 µM 5-aza-dC on GSTM5 protein expression. The anti-GSTM5 antibodies were Abnova H00002949-D01P. (**C**) Correlation between age and GSTM5 DNA methylation level in 50 bladder cancer tissues and 50 normal human urine pellets. The linear regression formula is on the bottom right (R^2^ = 0.1074), which was calculated by Microsoft Excel. (**D**) GSTM5 DNA methylation level in 50 bladder cancer tissues and 50 normal human urine pellets. The statistical results were analyzed by SigmaPlot. (**E**) Kaplan–Meier plot showing the overall survival probability of 404 bladder cancer patients. (**F**) Kaplan–Meier plot of the relapse-free survival probability of 187 bladder cancer patients.

In our previous study [24], GSTM5 mRNA expression was enhanced in 5637 bladder cancer cells following treatment with 10 µM 5-aza-dC; the DNA CpG methylation level of the GSTM5 promoter was also decreased from 84.6 to 61.5%. In the present study, 5-aza-dC administration also increased GSTM5 mRNA (Figure 2A) and protein (Figure 2B) expression in 5637 cells and to a greater degree in RT4 cells (Figure 2A,B).

In view of the inhibited gene activation and expression of GSTM5 caused by DNA CpG methylation, the DNA methylation level of GSTM5 was analyzed using tumors from patients with bladder cancer and urine pellets from healthy individuals with no previous history of bladder cancer. The characteristics of the study population are listed in Table 1. Though the mean age was different between patients and healthy subjects (Table 1), the correlation between age and GSTM5 DNA methylation level in the 100 volunteers was not statistically significant (Figure 2C). Though the sex distribution was also different between patients and healthy subjects, the GSTM5 DNA methylation level was no difference between male and female subjects (*p* = 0.7) (Table 2). When comparing 50 patients with 50 healthy subjects, GSTM5 DNA methylation level was significantly higher in bladder cancer tissues than in healthy urine pellets (Figure 2D). After the DNA methylation levels were classified into three levels, including low (<30%), medium (≥30% and <75%) and high (≥75%), the difference between healthy subjects and patients with bladder cancer also reached significance (*p* = 0.003) (Table 3). Differences between GSTM5 DNA methylation levels at various cancer stages were also analyzed, and the results also indicated a significant difference (Table 4). These findings suggest that hypermethylation of the GSTM5 gene represents an essential biomarker of bladder cancer, which results in the lower mRNA expression levels observed in Figure 1. Analysis of publicly available clinical survival data associated with GSTM5 expression (Kaplan–Meier Plotter, https://kmplot.com/analysis/, accessed on 20 November 2020) is displayed in Figure 2E,F. These data indicate that high GSTM5 expression may increase the probability of relapse-free survival in patients with bladder cancer (Figure 2F).

In addition, the GSTM1-null ratio was also analyzed in the 100 study volunteers by using the multiplex PCR method according to our previous study [24]. However, no significant differences were observed between normal subjects (62%) and those with bladder cancer (64%).

### 2.3. Biological Effects of GSTM1-5 Overexpression in Bladder Cancer Cells

**Figure 3:**
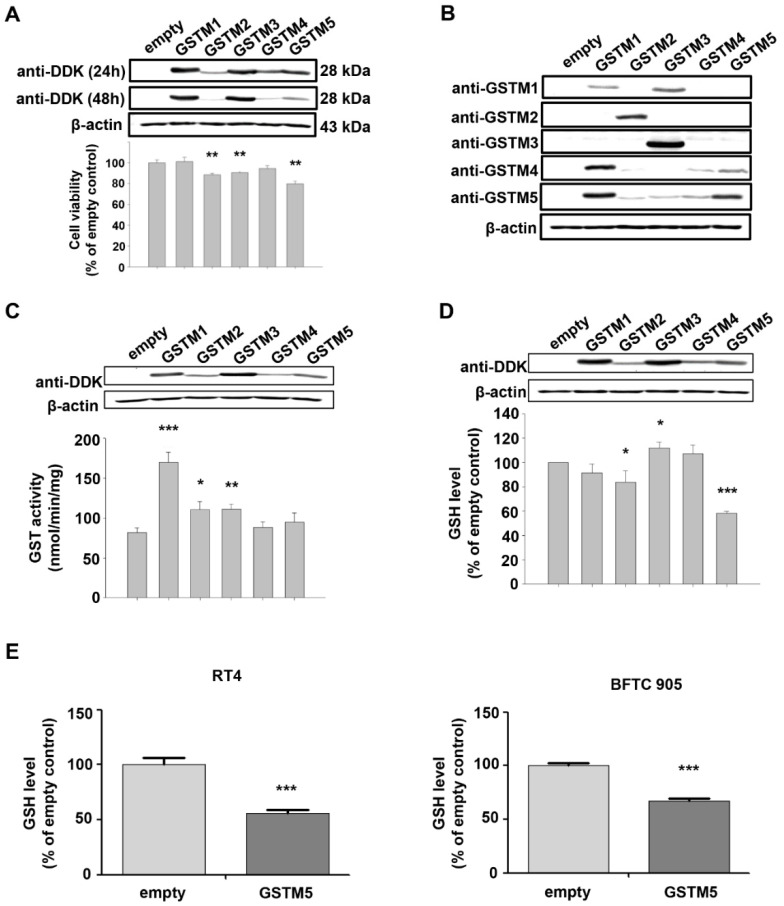
The expression and biological effects of GSTM1-5 by transient transfection expression. (**A**) The protein expression and cell viability after transfection. A total of 5637 cells were transfected with empty vectors or vectors with GSTM1-5 genes. After transfection for 24 h and 48 h, the proteins were detected by anti-DDK antibodies. Cell viability was analyzed after transfection for 32 h. (**B**) The protein expression and antibody characteristics. A total of 5637 cells were transfected with empty vectors or vectors with GSTM1-5 genes for 24 h; GSTM1~5 proteins were detected by their individual antibodies from GeneTex. (**C**) GST activity assay. A total of 5637 cells were transfected with empty vectors or vectors with GSTM1-5 genes for 24 h, the protein expression and GST activity were analyzed individually. (**D**) Cellular GSH level. A total of 5637 cells were transfected with empty vectors or vectors with GSTM1-5 genes for 24 h, the protein expression and GSH level (assayed by Cayman Chemical kit 703002) were analyzed. (**E**) Cellular GSH level in RT4 and BFTC 905 cells. After transient transfection for 24 h, cells were collected for GSH level detection (assayed by Cayman Chemical kit 703,002). All data are presented as the mean ± SE of three independent experiments. * *p* < 0.05; ** *p* < 0.01; *** *p* < 0.001.

**Figure 4:**
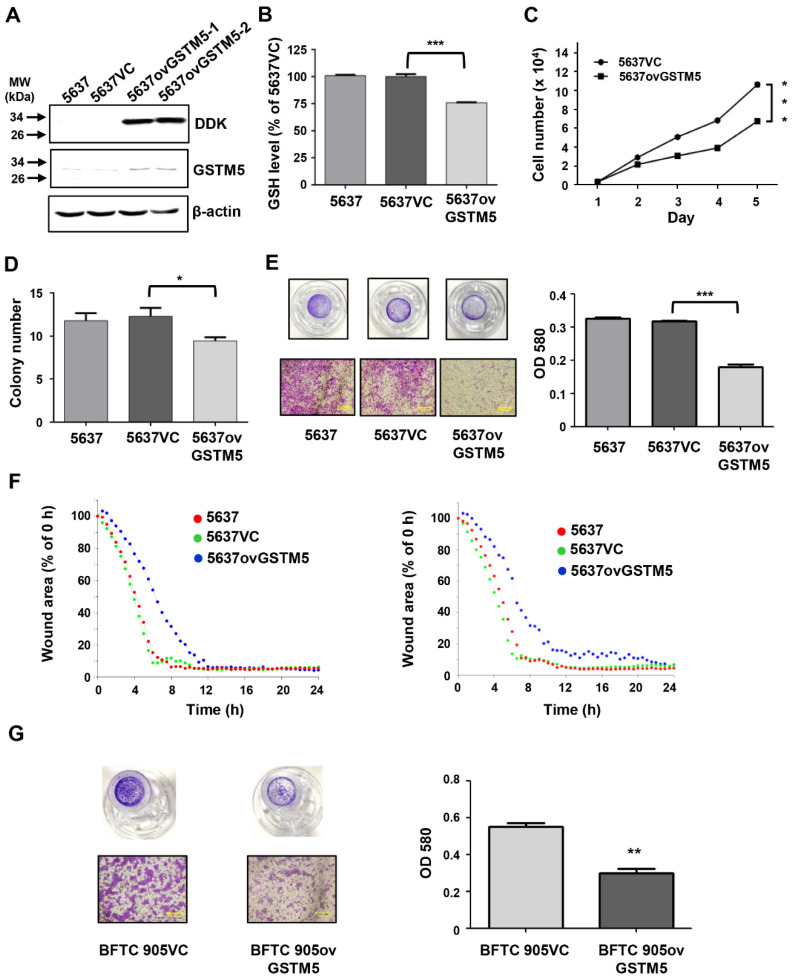
Bladder cancer cell proliferation and migration ability were decreased after GSTM5 overexpression. (**A**) The protein expression in 5637 cells with or without overexpression of empty vector (5637VC) or GSTM5 (5637ovGSTM5-1 and 5637ovGSTM5-2 were from 2 stably transfected colonies) was detected by Western blot. The anti-DDK antibodies (TA50011-100 from OriGene) and anti-GSTM5 antibodies (H00002949-D01P from Abnova) were used for detection. (**B**,**C**) GSTM5-overexpressed 5637 cells decreased intracellular GSH levels (**B**) and cell proliferation (**C**). The glutathione (GSH) levels were analyzed by a modified method described in Materials and Methods. (**D**) The number of colonies in cancer cells was decreased by GSTM5 overexpression. (**E**) The migration ability of 5637 cells was suppressed by GSTM5 overexpression by Transwell assay. Upper panel: Transwell insert with a circular membrane of 0.65 cm diameter. Lower panel: microscopic images: scale bar is 500 µm. All data are presented as the mean ± SE of three independent experiments. * *p* < 0.05; ** *p* < 0.01; *** *p* < 0.001. (**F**) Wound healing assay. Four different wound sites were recorded in each well for 24 h. The experiment was repeated once (right and left). Red line: 5637 cells, green line: 5637VC cells, blue line: 5637ovGSTM5 cells. (**G**) The migration capacity of BFTC 905 cells was inhibited by GSTM5 overexpression. Upper panel: Transwell inserts with a circular membrane of 0.65 cm diameter. Lower panel: microscopic images; scale bar is 500 µm.

In addition to GSTM5 and GSTM1, there are another 3 members in the GSTM family, including GSTM2-4. GSTM1~5 share 66 to 88% amino acid sequence similarity. GSTM2 is known to play a tumor suppressor role [21], and functional characteristics that may also be shared GSTM5 (Figure 1); therefore, the effects of GSTM1-5 on cell viability were compared by overexpressing these proteins in 5637 cells. GSTM1-5 were transiently overexpressed in 5637 cells and detected by anti-DDK flag (Figure 3A) or anti-GSTM1-5-specific antibodies (Figure 3B). The molecular weight of the overexpressed GSTMs was about 28–29 kDa, which is slightly greater than that of endogenous GSTMs (about 26 kDa). As shown in Figure 3A, the cell viability was significantly decreased in GSTM2, GSTM3 and GSTM5-overexpressed cells. Figure 3B indicates that anti-GSTM2 and -GSTM3 antibodies have a higher specificity than those against GSTM1, GSTM4 and GSTM5. Due to the nonspecific activity of the anti-GSTM5 antibody from GeneTex, another antibody from Abnova was used to generate the data presented in Figure 2B and Figure 4A. As the primary function of GSTs is to catalyze the conjugation of GSH to a wide variety of electrophilic substrates, the enzyme activity was also compared in 5637 cells overexpressing GSTM1-5, using CDNB as a substrate. The results suggested that GSTM1 possessed the greatest enzymatic activity (Figure 3C). Considering the differences in substrate binding affinity between GSTM1-5, the findings may not represent the real enzymatic activity of all cells overexpressing GSTMs; therefore, the cellular GSH level was also selected to assess the enzyme activity because the glutathione transferase consumes GSH as a result of this enzyme reaction. Figure 3D indicates that cellular GSH levels were decreased in cells overexpressing GSTM2 and GSTM5 but increased in GSTM3-overexpressed cells. Among them, GSTM5 overexpression depleted GSH levels to the greatest degree. The phenomenon of GSTM5 overexpression-reduced GSH cellular level was confirmed in two additional bladder cancer cell lines, RT4 and BFTC 905 (Figure 3E).

### 2.4. GSTM5 Overexpression Decreases Intracellular GSH Levels and Suppresses the Proliferation and Migration of Bladder Cancer Cells

The conjugation of GSH with various electrophilic compounds is supposed to decrease the cellular damage caused by the reactive compounds. However, the process also depletes cellular GSH, which is integral to other protective mechanisms in addition to glutathione transferase, including the glutathione peroxidase pathway. Since GSTM5 decreased GSH levels to a greater degree than the other GSTMs (Figure 3D), the potential effect of this GSTM5-induced GSH reduction in cellular activity was investigated. After stable overexpression, the protein levels of GSTM5 were significantly increased in 5637 cells (5637ovGSTM5) (Figure 4A). The cellular GSH concentration was also decreased in 5637ovGSTM5 cells compared with those transfected with the vector control (5637VC) (Figure 4B).

The cell viability was significantly decreased after transient GSTM5 expression (Figure 3A). In the stable cell lines, the effects of GSTM5 on the proliferative rate and colony formation ability were analyzed. The results indicated that the cell proliferation and the number of colonies in soft agarose were decreased by GSTM5 overexpression (Figure 4C,D). Moreover, the migration activity of 5637ovGSTM5 cells was significantly lower than that of 5637VC cells by Transwell assay (Figure 4E). Wound healing assays also showed that GSTM5 overexpression retarded the healing rate (Figure 4F) (Video S1). GSTM5 overexpression inhibited cell migration was also observed in BFTC 905 cells (Figure 4G). The results of the aforementioned functional assays indicate that GSTM5 plays a suppressor role in both bladder cancer proliferation and migration.

### 2.5. GSH Reverses GSTM5-Inhibited Cell Proliferation and Migration

**Figure 5:**
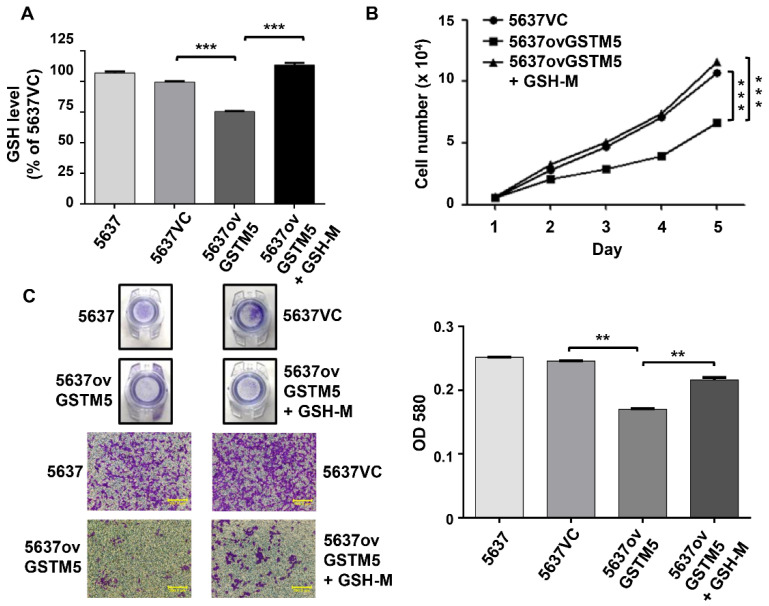
GSH supplementation restores the cell proliferation and migration ability of bladder cancer cells, overexpressing GSTM5. Four millimolar GSH-M-treated 5637ovGSTM5 cells showed an increase in (**A**) intracellular GSH levels, (**B**) cell proliferation and (**C**) cell migration. Upper panel: Transwell insert with a circular membrane of 0.65 cm diameter. Lower panel: microscopic images; scale bar is 500 µm. All data are presented as the mean ± SE of three independent experiments. ** *p* < 0.01; *** *p* < 0.001.

In the present study, GSTM5 was found to decrease cellular GSH levels and suppress cancer cell proliferation and migration. Therefore, to further elucidate whether GSH supplementation could restore cell proliferation and migration ability, 5637ovGSTM5 cells were treated with 4 mM GSH-M. Restoration of GSH levels was confirmed 24 h after GSH-M treatment (Figure 5A) and reversed the retardation of 5637ovGSTM5 cell proliferation (Figure 5B). In addition, the migration ability of 5637ovGSTM5 cells was significantly increased after GSH-M treatment (Figure 5C). Taken together, these data suggest that the GSTM5-induced suppression of cancer cell proliferation and migration were reversed by intracellular GSH supplementation.

### 2.6. BSO Administration Decreases Intracellular GSH and Suppresses the Proliferation and Migration of 5637 Bladder Cancer Cells

**Figure 6:**
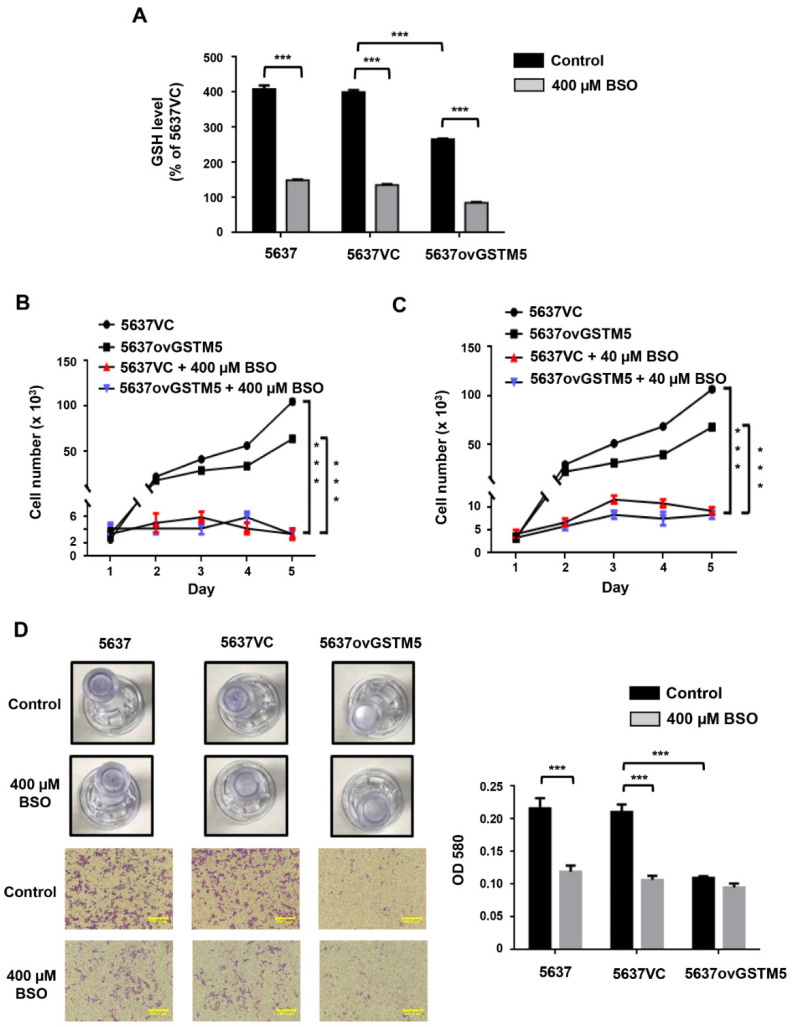
BSO decreased cell migration and proliferation. (**A**) 400 µM buthionine sulfoximine (BSO) treatment for 24 h decreased the intracellular GSH concentration in cells. (**B**) 400 µM BSO blocked cell proliferation. The medium was changed once on day 4. (**C**) 40 µM BSO also decreased cell proliferation. The medium was changed every day. (**D**) Effect of 400 µM BSO on cell migration of 5637 (left), 5637VC (middle) and 5637ovGSTM5 (right) cells. Upper panel: Transwell insert with a circular membrane of 0.65 cm diameter. Lower panel: microscopic images; scale bar is 500 µm. All t data are presented as the mean ± SE of three independent experiments. *** *p* < 0.001.

BSO is an inhibitor of the glutamate-cysteine ligase catalytic subunit, which is the key enzyme for GSH biosynthesis. After treatment with 400 µM BSO for 24 h, intracellular GSH levels were decreased (Figure 6A), and proliferation was almost arrested (Figure 6B) in both 5637VC and 5637ovGSTM5 cells. Since 400 µM BSO had such a marked impact on proliferation after treatment for 2 days, a decreased dose of 40 µM was used for proliferation assay again (Figure 6C). Treatment with 40 µM BSO also significantly decreased the rates of 5637VC and 5637ovGSTM5 cell proliferation (Figure 6C). In cell migration assay, BSO significantly reduced cell migration capacity of 5637 and 5637VC cells, but not 5637ovGSTM5 cells because the migration ability of the latter was originally on the low side (Figure 6D).

### 2.7. Cell Adhesion Capacity Is Decreased in GSTM5-Overexpressing Cells

**Figure 7:**
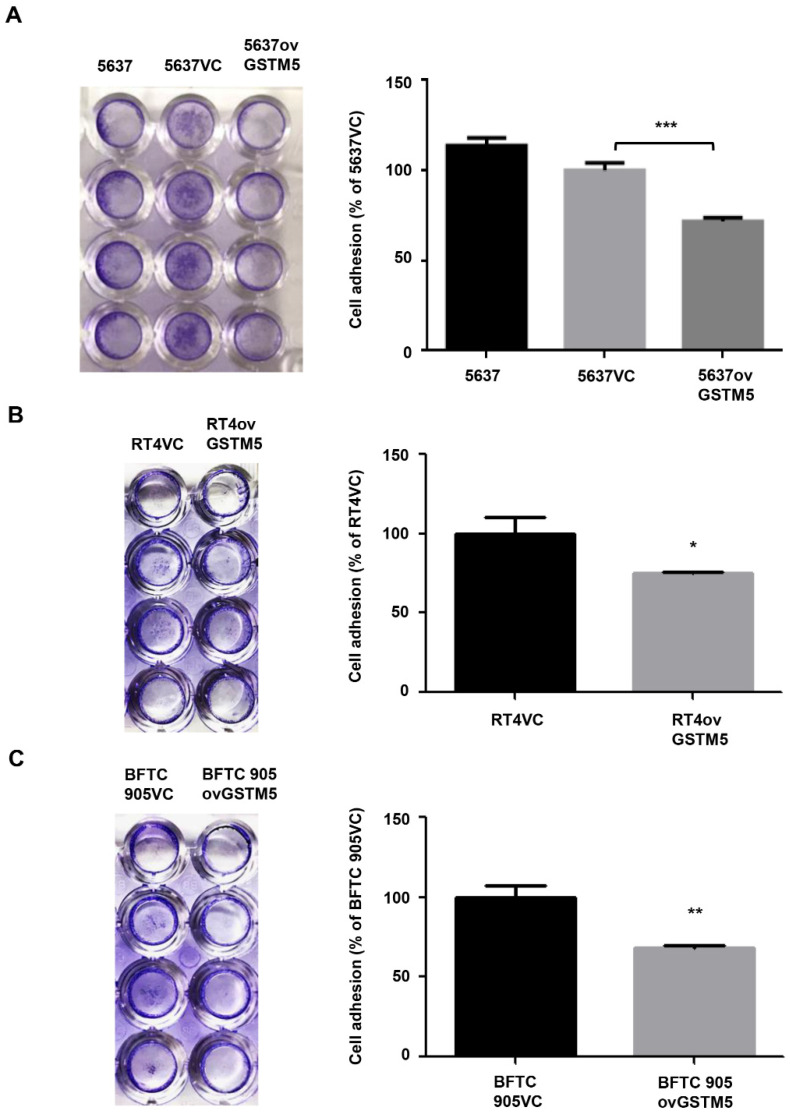
Cell adhesion analysis. (**A**) The adhesion capacity of 5637, (**B**) RT4, and (**C**) BFTC 905 cells. Each VC cell was set as 100%. The data are presented as the mean ± SE of three independent experiments. * *p* < 0.05, ** *p* < 0.01, *** *p* < 0.001.

Cell adhesion to the extracellular matrix is one of the important characteristics in tumor cell invasion [25]. Thus, this functional assay was performed to determine the effects of GSTM5 therein. Figure 7A shows that 5637ovGSTM5 cells exhibited a reduced ability of cell adhesion to fibronectin compared with 5637VC cells. This inhibition effect was also observed in RT4 (Figure 7B) and BFTC 905 cells (Figure 7C).

### 2.8. Effects of GSTM5 Overexpression on the Cytotoxic Sensitivity of Doxorubicin, Cisplatin and Mitomycin C

**Figure 8:**
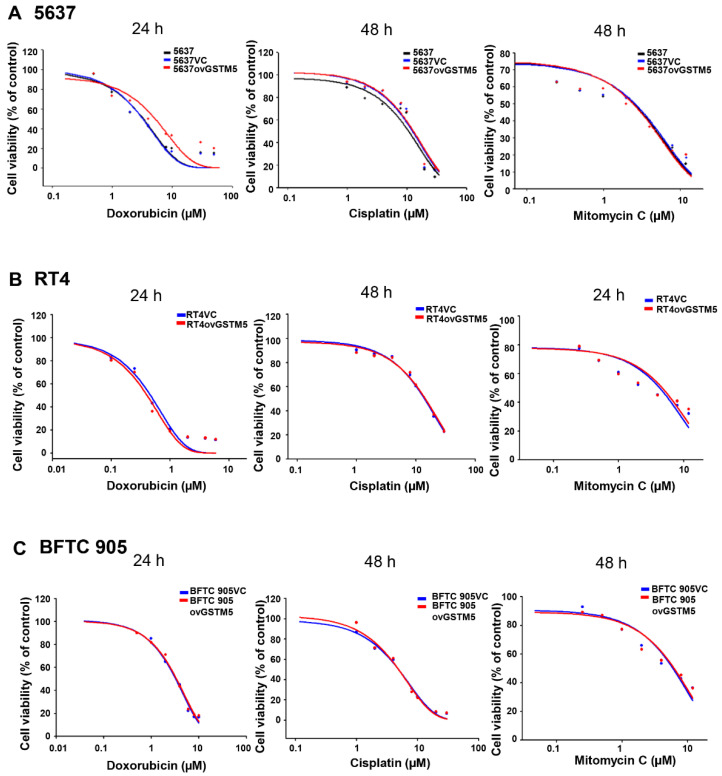
GSTM5 overexpression does not significantly alter the sensitivity of cells to doxorubicin, cisplatin and mitomycin C. (**A**) The drug sensitivity of 5637, 5637VC and 5637ovGSTM5 cells (**B**) drug sensitivity of RT4VC and RT4ovGSTM5. (**C**) Drug sensitivity of BFTC 905VC and BFTC 905ovGSTM5. Cells were analyzed in a wide-range drug concentration by MTT assay. The individual drug treatment times are displayed above each data.

Overexpression of GSTM5 was found to decrease cancer cell proliferation and migration by decreasing cellular GSH levels. Therefore, we want to know whether cancer cells overexpressing GSTM5 induce resistance to anticancer drugs or not. The results indicated that the drug sensitivity of 5637ovGSTM5 in response to cisplatin and mitomycin C was not altered and that sensitivity to doxorubicin was marginally decreased (Figure 8A). In addition, the drug sensitivity was also analyzed in RT4 and BFTC 905 cells. It indicated that no obvious change between vector control-transfected and GSTM5 overexpressing RT4 (Figure 8B) and BFTC 905 (Figure 8C) cells. These findings suggest that overexpression of GSTM5 protein did not affect the susceptibility of bladder cancer cells to cisplatin and mitomycin C, which are commonly used anticancer drugs for bladder cancer treatment.

## 3. Discussion

The present study revealed that low GSTM5 gene expression and high GSTM5 DNA methylation level tended to bladder cancer (Figure 1 and Figure 2D) and that patients with bladder cancer and high GSTM5 expression had a longer relapse-free survival (Figure 2F). In vitro assays demonstrated that GSTM5 inhibited cell proliferation (Figure 4C), colony formation (Figure 4D), cell migration (Figure 4E–G) and fibronectin adhesion (Figure 7). Furthermore, GSTM5-induced GSH decrease was found to play a role in the inhibition of cell proliferation and migration. GSH, a small molecule with a molecular weight of 307, is a cellular metabolite found in the majority of organisms. GSH is an antioxidant whose functions are dependent on the thiol group of cysteine residues, and in its reduced form, is a marketed drug for maintaining normal liver function. Furthermore, GSH also manifests other biologic functions, especially those associated with cancer cells. For example, increased GSH levels promoted cell proliferation in HepG2 cells [26] and cell metastasis in B16 melanoma cells [27]. The information elucidates that intracellular GSH concentration affects cancer cell function and plays a pivotal role in cancer characteristics.

Redox homeostasis is important for both normal and cancer cells. The cells will undergo death following redox imbalance, where the levels of reactive oxygen species exceed the intrinsic antioxidant capacity. In cancer cells, elevated GSH level not only counteracts elevated oxidative stress but also contributes to chemotherapeutic resistance [28]. For example, GSH mediates Nrf2-induced boningmycin resistance in A549 and HepG2 cancer cells [29]. In the present study, the GSTM5-reduced GSH level inhibited the proliferation of bladder cancer cells (Figure 4C) but did not affect sensitivity to cisplatin and mitomycin C (Figure 8). Although the GSH conjugation function of GSTs can neutralize electrophilic endogenous and xenobiotic compounds by introducing a GSH molecule into them [30], GSTM5 overexpression accompanied by GSH decreasing did not affect the cytotoxic sensitivity of cisplatin and mitomycin C. This suggests that GSTM5 induction may be a potential strategy for bladder cancer treatment without inducing significant resistance to cisplatin and mitomycin C. GSTM5 overexpression slightly increased resistance to doxorubicin in 5637 cells (Figure 8A), increasing the IC_50_ from 3.2 µM (5637VC) to 5.2 µM (5637ovGSTM5). However, no change in doxorubicin sensitivity was observed in RT4 and BFTC 905 cells (Figure 8B,C). Therefore, the influence of GSTM5 on doxorubicin sensitivity is different between diverse cancer cells. It is still unclear what molecular mechanism contributes to the difference, but similar results have been reported in the study of GSTP1. In stable transfection of laryngeal carcinoma, HEp2 cells with human GSTP1 resulted in a 3-fold increase in doxorubicin resistance, compared with that of the control cells [31]. However, in ovarian cancer cells, stable GSTP1 knockdown did not significantly influence the IC_50_ value of doxorubicin [32]. According to the aforementioned reports and our results, it suggests that the influence of GSTs on drug sensitivity should be analyzed in individual cell lines.

The anticancer role of GSTs has been reported in some studies. In non-small cell lung cancer, GSTM2 can inhibit cell migration [21]. In hepatocellular carcinoma cells, GSTZ1 also serves as a tumor suppressor [33]. To date, several studies have reported a correlation between GSTM5 and cancer, though the direct effects of GSTM5 on tumor cells remain unclear. For example, the gene expression of GSTM5 is lower in tumor tissues than in normal tissues in Barrett’s adenocarcinoma [23], GSTM5 expression is downregulated in breast and prostate cancer [34]. GSTM5 expression was also shown to be a positive biomarker for survival in ovarian serous cystadenocarcinoma [35]. The aforementioned reports and the present study suggest that GSTM5 expression and its DNA methylation levels may act as biomarkers for bladder cancer progression. To the best of our knowledge, the current study is the first to directly indicate that GSTM5 plays an anticancer role and that the GSTM5-reduced GSH mediates an important mechanism for this anticancer effect. However, it is still unknown how GSTM5 reduces GSH, which will be investigated in future metabolite analysis.

There is one major limitation in the current study that should be addressed in future investigations. Since the collection of sufficient normal bladder tissues via regular surgery was somewhat challenging; therefore, we decided to use urine pellets of healthy individuals as the alternative target specimens of comparative control. Although urine pellets may contain not only bladder-originated cells, however, many previous studies have demonstrated the appropriateness of urine pellets in DNA methylation analysis of bladder cancers [36,37,38]. Therefore, in future studies, the GSTM5 DNA methylation status will be compared between paired bladder tissues and urine pellets.

## 4. Materials and Methods

### 4.1. Extraction of Genomic DNA from Human Bladder Tumors, Human Urine Pellets and Cultured Cell Lines

Bladder tumor samples (*n* = 50) from patients with bladder cancer and urine pellets (*n* = 50) from nonbladder cancer subjects were collected. All subjects gave their informed consent for inclusion before sample collection. The study was conducted in accordance with the Declaration of Helsinki 2013, and the study protocol was reviewed and approved by the institutional review board (IRB) of Ditmanson Medical Foundation Chiayi Christian Hospital (Chiayi, Taiwan). The approval number is CYCH-IRB 103070 (approved on 5 Jan 2015). DNA extraction of bladder tumor samples, urine pellets and culture cells followed the instructions of the DNA extraction kit (Geno Plus Genomic DNA extraction miniprep system, Viogene, New Taipei City, Taiwan).

### 4.2. Bisulfite Conversion of Genomic DNA and Analysis of the DNA Methylation Level in Human Samples

Bisulfite conversion was performed using the EZ DNA methylation-gold kit (Zymo Research, Irvine, CA, USA). Five hundred nanograms of genomic DNA was reacted with sodium bisulfite following the kit instructions. After bisulfite conversion, the selected *GSTM5* DNA promoter region was PCR amplified using bisulfite-specific primers: 5′- TTTAGGGYGGGTTGGGGAAGTTGG -3′ (forward) and 5′- AAAAAAAACRATCCCTAAAACCAATCCTACAACTAC -3′ (reverse), which amplified −209 to 198 bp of the human *GSTM5* gene (amplicon size 407 bp). Next, the PCR products were subcloned into the T&A cloning vectors, which were transformed into competent cells. To determine the CpG methylation status of the 5′ CpG island of *GSTM5*, 10 clones of each sample were randomly picked for sequencing.

### 4.3. Cell Culture and RT–PCR

Three human bladder cancer cell lines, RT4, BFTC 905 and 5637, were purchased from Bioresource Collection and Research Center (Hsinchu, Taiwan). RT4 cells were cultured in McCoy’s 5a medium with 10% fetal bovine serum, 1% penicillin and 1% streptomycin, BFTC 905 and 5637 cells were cultured in RPMI 1640 medium with the same additional agents [24]. After washing the cells with PBS and lysing the cells with TRIzol reagent (Thermo Fisher Scientific, Waltham, MA, USA), total RNA was extracted according to the manufacturer’s instructions. Reverse transcription (RT) was performed on 2 μg of total RNA by 1.5 μM random hexamer and RevertAid™ reverse transcriptase (Fermentas, Waltham, MA, USA). Then, 1/20 volume of the reaction mixture was used for PCR with human *GSTM5*-specific primers (5′ ATGCCCATGACACTGGGGTACTG 3′, 5′ CCATGTGGTTATCCATAACCTGG 3′, product size 328 bp) and *GAPDH*-specific primers (5′CAAGGTCATCCATGACAACTTTG3′, 5′GTCCACCACCCTGTTGCTGTAG3′, product size 496 bp). The PCR products were analyzed by 1–2% agarose gel electrophoresis.

### 4.4. Western Blot

Total lysates of 5637 cells, empty vector-transfected 5637 cells (5637VC), and GSTM5-overexpressed 5637 cells (5637ovGSTM5) were extracted by PRO-PREP protein extraction solution (iNtRON Biotechnology, Burlington, MA, USA). Equal amounts of protein (30 μg) were separated by 8% SDS-polyacrylamide gels electrophoresis and transferred to polyvinylidene difluoride membranes. Immunodetection was performed using antibodies against human GSTM1~5 (GTX100298, GTX47111, GTX65535, GTX32637, GTX108776, GeneTex, Irvine, CA, USA), against GSTM5 (H00002949-D01P, Abnova, Taipei, Taiwan) and against β-actin (A5441, Sigma-Aldrich, St. Louis, MO, USA) as an internal control. Mouse or rabbit IgG antibodies coupled to horseradish peroxidase (Jackson ImmunoResearch, West Grove, PA, USA) were used as the secondary antibodies. Protein expression was visualized by Luminol/Enhancer solution (Thermo Scientific, Waltham, MA, USA) and a luminescence detector.

### 4.5. GSTMs Overexpression in 5637 Cells

GSTM1~5 expression vectors were purchased from Origene, Rockville, MD, USA (RC223332, RC210718, RC201013, RC202097, RC208900) with Myc-DDK tagged. A total of 5637 cells were cultured in a 6-well plate with a density of 3 × 10^5^ cells/well and transfected the next day. The transfection reagent was PolyJet in vitro DNA transfection reagent (SignaGen Laboratories, Frederick, MD, USA) in the combination of 1 µg plasmid DNA and 3 µL PolyJet. After incubation for 6 h, the transfection medium was replaced by a fresh medium without DNA and PolyJet. For stable clone selection, the transfected cells were subcultured in 10 cm dishes with 800 µg/mL antibiotic G418 (GoldBio, St Louis, MO, USA). After 5 days, single colonies were picked up individually and seeded into a 24-well plate for amplification. The stable transfection cell lines were kept in a culture medium with 400 µg/mL G418.

### 4.6. GSTM5 Overexpression in RT4 and BFTC 905 Cells

Cells were cultured in 10 cm dishes with 80% confluence for transfection. The GSTM5 expression vector with Myc-DDK tagged and control vector was from Origene. Cells in one dish were incubated with the combination of 5 µg plasmid DNA and 15 µL PolyJet. After incubation for 24 h, the cells were collected for GSH level assay (6 × 10^5^ cells/sample) and seeded in the density of 6 × 10^4^ cells/Transwell/24-well for migration assay, 2 × 10^4^ cells/well/96-well for fibronectin adhesion assay, 3 × 10^4^ cells/well/24-well (BFTC 905) or 6 × 10^4^ cells/well/24-well (RT4) for drug cytotoxicity assay

### 4.7. GST Activity Assay

The detection method of GST activity was modified from a GST activity assay kit from Cayman Chemical, Ann Arbor, MI, USA (item no. 703,302). After cell transfection for 24 h, cells were collected in GST store buffer (100 mM KH_2_PO_4_ (J.T.Baker, Radnor, PA, USA), 2 mM EDTA (Thermo Fisher Scientific)) and sonicated on ice. After centrifugation at 10,000× *g* for 10 min, 4 ℃, then the supernatant was further centrifuged at 100,000× *g* for 1 h, 4 ℃. The supernatant (cytosol fraction) was analyzed for GST activity in a 96-well plate. Twenty µL supernatant was added into a well with assay buffer (75 mM KH_2_PO_4_ (J.T.Baker), 0.075% Triton X-100 (J.T.Baker), 2 mM *L*-glutathione-reduced form (Alfa Aesar, Haverhill, MA, USA) on ice. After adding substrate 1-chloro-2,4-dinitrobenzene (CDNB) (Alfa Aesar) and mixing well, the OD340 nm values were read by a spectrophotometer at 25 ℃ for 20 min with 1 min interval. GST activity was calculated as [(OD340/min)/0.00503 µM^−1^] × [0.2 mL/0.02 mL] = nmol/min/mL, then divided by protein concentration (mg/mL) to get the final GST activity (nmol/min/mg).

### 4.8. Cellular GSH Quantification

The detection of intracellular glutathione levels was using a glutathione assay kit (Cayman Chemical item no. 703,002) or a modified method from this kit. For the kit assay, one-well cells of a 6-well plate were collected with 1 mL of cell homogenization buffer (50 mM 2-(*N*-morpholino)ethanesulfonic acid (MES) (Sigma-Aldrich), 1 mM EDTA, pH 6.0) and sonicated on ice. In the modified assay, two 6 cm plates of cells were collected with 0.5 mL of the same cell homogenization buffer and sonicated on ice. After centrifugation at 10,000× *g* at 4 °C for 15 min, the supernatant was collected in a 1.5 mL tube. The cell supernatants were mixed with 5% metaphosphoric acid (MPA) (Sigma-Aldrich) for 5 min for deproteination and then centrifuged at RT for 5 min. One milliliter of supernatant was mixed with 50 µL of 4 M triethanolamine (Sigma-Aldrich) for pH adjustment. GSH standards (1000, 500, 250, 125, 62.5, 31.25 and 15.625 µM) with serial dilutions were prepared for making standard curve plots. Next, 150 μL of cocktail reagent (0.1 M MES, 0.025 M phosphoric acid, 0.5 mM EDTA, 20 µM 5,5′-dithiobis(2-nitrobenzoic acid) (DTNB) (Sigma-Aldrich)) and 50 μL of sample/standard were added to 96-well plates. After the 96-well plate was incubated at room temperature for 25 min, the absorbance was read at 405 nm. For supplementation of cellular GSH, glutathione-reduced ethyl ester (GSH-M, Sigma-Aldrich) was added to the cell medium for 24 h before assay.

### 4.9. Cell Proliferation Assay

Bladder cancer cells were seeded in 12-well plates at 6000 cells per well in RPMI-1640 medium with 400 µg/mL G418. The medium with or without 4 mM GSH-M, or 40 µM buthionine sulfoximine (BSO) (Sigma-Aldrich), was changed every day. Cells were trypsinized and counted using a hemocytometer every day.

### 4.10. Colony Formation Assay

Before cell seeding, each well was covered with 0.5% agarose (Uni-Onward, New Taipei City, Taiwan) at 37 ℃ for 30 min. Bladder cancer cells (2 × 10^4^ cells/well) were seeded into six-well culture plates in 0.3% agarose and incubated for 2 weeks to allow colony formation. The medium (2 mL/well) was changed every 3 days. The number of colonies from three independent experiments was counted using ImageJ, version 1.48 (NIH, Bethesda, MD, USA).

### 4.11. Migration Assay

Migration assays were performed using 24-well Transwell chambers with 8 µm pore membrane (Wuxi NEST Biotechnology, Wuxi, Jiangsu, China). Bladder cancer cells were seeded in the upper chamber at 3 × 10^4^ cells/well (5637) or 6 × 10^4^ cells/well (BFTC 905) in 0.1 mL of RPMI 1640 serum-free medium. Medium with 10% FBS was placed in the bottom well (0.5 mL/well). After incubation for 24 h at 37 °C in an atmosphere containing 5% CO_2_, the medium was discarded. Then cells were fixed in 4% formaldehyde (Sigma-Aldrich) at room temperature for 2 min, and then in methanol for 15 min. After PBS wash, cells were stained with 0.5% crystal violet (Sigma-Aldrich) for 5 min. After PBS wash, cells on the inside surface of the Transwell were removed by a cotton swab. The chambers were photographed, and stained cells were dissolved with DMSO for 10 min and then detected for the OD at 580 nm.

### 4.12. Wound Healing Assay

Cells were seeded in 6-well plates. After the cells reached confluence, a wound was made with a 200-µL plastic tip in each well. The wells were then washed twice with PBS to remove cell debris and then incubated with a culture medium. The wound areas were automatically monitored for 24 h by taking a photograph every 30 min in an augmented microscope (Lionheart, BioTek, Winooski, VT, USA). The wound areas were automatically analyzed by computer, and the wound healing curve was performed based on the area change over time. The wound area of each condition was presented as 100 at the beginning. The values are the mean of four wounds in each well.

### 4.13. Cell Adhesion Assay

The 96-well plates were precoated with fibronectin (5 µg/cm^2^) (Corning, corning, NY, USA) at 37 °C for 2 h. A total of 8 × 10^3^ cells/well (5637) or 2 × 10^4^ cells/well (BFTC 905) or 8 × 10^3^ cells/well (RT4) in serum-free RPMI 1640 were seeded into each well and incubated at 37 °C for 2 h (5636) or 3.5 h (BFTC 905 and RT4). After incubation, nonattached cells were washed out by PBS, and then attached cells were fixed in 4% formaldehyde at room temperature for 30 min. Subsequently, cells were stained with 0.5% crystal violet for 1 min. After PBS wash, the plate was photographed, and cells were dissolved in DMSO. The absorbance at 580 nm was detected.

### 4.14. MTT (3-(4,5-Dimethylthiazol-2-yl)-2,5-Diphenyltetrazolium Bromide) Assay

Cell viability was detected by the MTT assay. The 5637 cells were seeded at 3 × 10^4^ cells/well into 24-well plates for 24 h and then treated with doxorubicin (Sigma-Aldrich) for 24 h or treated with mitomycin C (Sigma-Aldrich) or cisplatin (Sigma-Aldrich) for 48 h. Twenty-five microliters of MTT (50 mg/mL) (AK Scientific, Union City, CA, USA) was added to each well and incubated for 90 min in a 5% CO_2_ incubator. After incubation, discarded the medium and 500 µL of DMSO was added to each well and mixed well by micropipette. One hundred microliters of the suspension were transferred to a 96-well plate, and the OD values were read at 595 nm.

### 4.15. Statistical Analysis

Statistical differences were analyzed by one-way analysis of variance followed by Student’s *t*-test. The statistics in human samples were analyzed by SPSS for Windows version 21.0 (IBM Corp., Armonk, NY, USA) except noted in the figure legend. All statistics in the cell line assay were calculated using GraphPad Prism software, version 5.0 (GraphPad Prism software, La Jolla, CA, USA). Numerical data are expressed as the mean ± standard error (SE) from three independent experiments.

## 5. Conclusions

**Figure 9:**
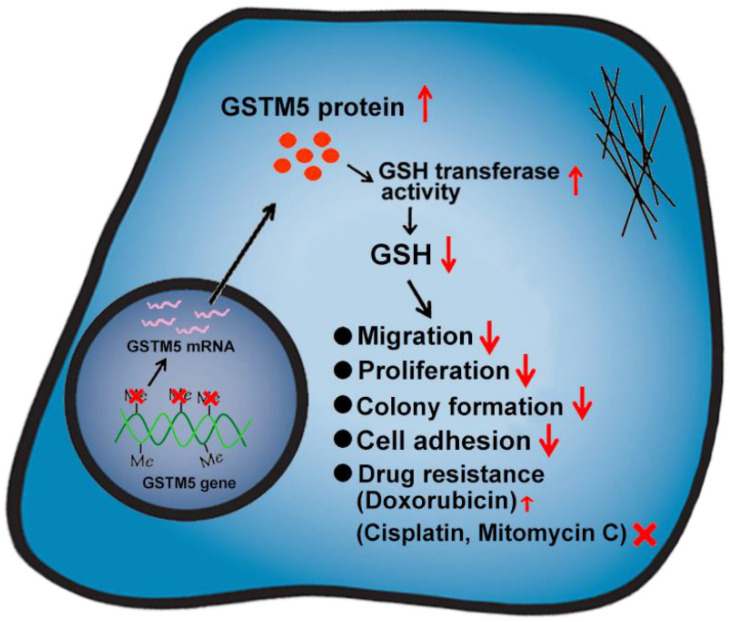
Schematic of GSTM5-inhibited bladder cancer. Demethylation of the GSTM5 gene CpG island promotes gene activation and mRNA expression. Increased GSTM5 protein expression reduces the intracellular GSH concentration. Decreased GSH levels inhibit cell migration, proliferation, colony formation and cell adhesion. In chemotherapeutic drug resistance, decreased GSH increases doxorubicin resistance slightly but has no effect on cisplatin and mitomycin C.

To the best of our knowledge, the present study was the first to demonstrate that the anticancer role of GSTM5 in bladder cancer and that the decreased levels of GSH play an important mediator of this function (Figure 9). As high GSTM5 expression was associated with a higher probability of relapse-free survival, in the future, increasing GSTM5 expression may be a consideration for bladder cancer therapy, and GSTM5 DNA methylation level may be a useful biomarker for patients with bladder cancer.

## Figures and Tables

**Table 1:** The age and sex of 50 patients with bladder cancer and 50 healthy individuals.

Characteristics	Bladder Cancer	Healthy Individual
Age (min/max)	28/92	20/81
Mean of age	73.16	35.42
Sexuality (male/female)	32/18	11/39

**Table 2:** Comparison of the sex differences between groups and GSTM5 DNA methylation levels.

Sample Group	Male	Female	*p*-Value
Group			
Bladder cancer number (%)	32 (74.42)	18 (31.58)	<0.001
Healthy individual number (%)	11 (25.58)	39 (68.42)	
All samples			
Methylation level (%)	61.17 ± 22.62	59.43 ± 22.32	0.700

**Table 3:** GSTM5 gene methylation levels of 50 patients with bladder cancer and 50 healthy individuals. The methylation level is divided into 3 classes (low, medium and high).

Three-Tier Classification of Methylation Level	Bladder Cancer Number (%)	Healthy Individual Number (%)	*p*-Value
Methylation levels (%)			0.003
Low (<30)	0 (0)	10 (20)	
Medium (30 ≦ x < 75)	32 (64)	28 (56)	
High (≧75)	18 (36)	12 (24)	

**Table 4:** The correlation between GSTM5 gene methylation levels and bladder cancer stages in 100 subjects. The methylation level is divided into 3 classes (low, medium and high).

Cancer Stage	Methylation Levels			*p*-Value
	Low (<30) Number (%)	Medium (30 ≦ x < 75) Number (%)	High (≧75) Number (%)	
Bladder status				0.031
Normal	10 (100)	28 (47.67)	12 (40)	
Stage 0is	0 (0)	9 (15)	2 (6.67)	
Superficial	0 (0)	17 (28.33)	11 (36.67)	
Infiltrating	0 (0)	6 (10)	5 (16.67)	
Total	10 (100)	60 (100)	30 (100)	

## Data Availability

The data and materials used in the current study are available from the corresponding author on reasonable request.

## Supplementary Materials

The following are available online at https://www.mdpi.com/1422-0067/22/6/3056/s1, Video S1: Wound healing assay. Time-lapse images were recorded from 0 to 14 h after wound production. The real time is presented in the right-upper corner. Left: 5637 cells, middle: 5637VC cells, right: 5637ovGSTM5 cells.

## References

[1] Siegel R.L., Miller K.D., Jemal A. (2019). Cancer statistics, 2019. CA Cancer J. Clin..

[2] Antoni S., Ferlay J., Soerjomataram I., Znaor A., Jemal A., Bray F. (2017). Bladder Cancer Incidence and Mortality: A Global Overview and Recent Trends. Eur. Urol..

[3] Stenehjem D.D., Tran D., Nkrumah M.A., Gupta S. (2018). PD1/PDL1 inhibitors for the treatment of advanced urothelial bladder cancer. Onco Targets Ther..

[4] Lower G.M. (1982). Concepts in causality: Chemically induced human urinary bladder cancer. Cancer.

[5] Brennan P., Bogillot O., Cordier S., Greiser E., Schill W., Vineis P., Lopez-Abente G., Tzonou A., Chang-Claude J., Bolm-Audorff U. (2000). Cigarette smoking and bladder cancer in men: A pooled analysis of 11 case-control studies. Int. J. Cancer.

[6] Straif K., Benbrahim-Tallaa L., Baan R., Grosse Y., Secretan B., El Ghissassi F., Bouvard V., Guha N., Freeman C., Galichet L. (2009). A review of human carcinogens—Part C: Metals, arsenic, dusts, and fibres. Lancet Oncol..

[7] Jou Y.C., Wang S.C., Dai Y.C., Chen S.Y., Shen C.H., Lee Y.R., Chen L.C., Liu Y.W. (2019). Gene expression and DNA methylation regulation of arsenic in mouse bladder tissues and in human urothelial cells. Oncol. Rep..

[8] Burger M., Catto J.W., Dalbagni G., Grossman H.B., Herr H., Karakiewicz P., Kassouf W., Kiemeney L.A., La Vecchia C., Shariat S. (2013). Epidemiology and risk factors of urothelial bladder cancer. Eur. Urol..

[9] Oakley A. (2011). Glutathione transferases: A structural perspective. Drug Metab. Rev..

[10] Hayes J.D., Strange R.C. (2000). Glutathione S-transferase polymorphisms and their biological consequences. Pharmacology.

[11] Engel L.S., Taioli E., Pfeiffer R., Garcia-Closas M., Marcus P.M., Lan Q., Boffetta P., Vineis P., Autrup H., Bell D.A. (2002). Pooled analysis and meta-analysis of glutathione S-transferase M1 and bladder cancer: A HuGE review. Am. J. Epidemiol..

[12] Kang H.W., Song P.H., Ha Y.S., Kim W.T., Kim Y.J., Yun S.J., Lee S.C., Choi Y.H., Moon S.K., Kim W.J. (2013). Glutathione S-transferase M1 and T1 polymorphisms: Susceptibility and outcomes in muscle invasive bladder cancer patients. Eur. J. Cancer.

[13] Matic M., Pekmezovic T., Djukic T., Mimic-Oka J., Dragicevic D., Krivic B., Suvakov S., Savic-Radojevic A., Pljesa-Ercegovac M., Tulic C. (2013). GSTA1, GSTM1, GSTP1, and GSTT1 polymorphisms and susceptibility to smoking-related bladder cancer: A case-control study. Urol. Oncol..

[14] Chuang J.J., Dai Y.C., Lin Y.L., Chen Y.Y., Lin W.H., Chan H.L., Liu Y.W. (2014). Downregulation of glutathione S-transferase M1 protein in N-butyl-N-(4-hydroxybutyl)nitrosamine-induced mouse bladder carcinogenesis. Toxicol. Appl. Pharmacol..

[15] Pearson W.R., Vorachek W.R., Xu S.J., Berger R., Hart I., Vannais D., Patterson D. (1993). Identification of class-mu glutathione transferase genes GSTM1-GSTM5 on human chromosome 1p13. Am. J. Hum. Genet..

[16] Bhattacharjee P., Paul S., Banerjee M., Patra D., Banerjee P., Ghoshal N., Bandyopadhyay A., Giri A.K. (2013). Functional compensation of glutathione S-transferase M1 (GSTM1) null by another GST superfamily member, GSTM2. Sci. Rep..

[17] Yu K.D., Fan L., Di G.H., Yuan W.T., Zheng Y., Huang W., Chen A.X., Yang C., Wu J., Shen Z.Z. (2010). Genetic variants in GSTM3 gene within GSTM4-GSTM2-GSTM1-GSTM5-GSTM3 cluster influence breast cancer susceptibility depending on GSTM1. Breast Cancer Res. Treat..

[18] Weng M.W., Hsiao Y.M., Chiou H.L., Yang S.F., Hsieh Y.S., Cheng Y.W., Yang C.H., Ko J.L. (2005). Alleviation of benzo[a]pyrene-diolepoxide-DNA damage in human lung carcinoma by glutathione S-transferase M2. DNA Repair.

[19] Tang S.C., Sheu G.T., Wong R.H., Huang C.Y., Weng M.W., Lee L.W., Hsu C.P., Ko J.L. (2010). Expression of glutathione S-transferase M2 in stage I/II non-small cell lung cancer and alleviation of DNA damage exposure to benzo[a]pyrene. Toxicol. Lett..

[20] Tang S.C., Wu M.F., Wong R.H., Liu Y.F., Tang L.C., Lai C.H., Hsu C.P., Ko J.L. (2011). Epigenetic mechanisms for silencing glutathione S-transferase m2 expression by hypermethylated specificity protein 1 binding in lung cancer. Cancer.

[21] Tang S.C., Wu C.H., Lai C.H., Sung W.W., Yang W.J., Tang L.C., Hsu C.P., Ko J.L. (2013). Glutathione S-transferase mu2 suppresses cancer cell metastasis in non-small cell lung cancer. Mol. Cancer Res. MCR.

[22] Ibragimova I., Dulaimi E., Slifker M.J., Chen D.Y., Uzzo R.G., Cairns P. (2014). A global profile of gene promoter methylation in treatment-naive urothelial cancer. Epigenetics.

[23] Peng D.F., Razvi M., Chen H., Washington K., Roessner A., Schneider-Stock R., El-Rifai W. (2009). DNA hypermethylation regulates the expression of members of the Mu-class glutathione S-transferases and glutathione peroxidases in Barrett’s adenocarcinoma. Gut.

[24] Wang S.C., Huang C.C., Shen C.H., Lin L.C., Zhao P.W., Chen S.Y., Deng Y.C., Liu Y.W. (2016). Gene Expression and DNA Methylation Status of Glutathione S-Transferase Mu1 and Mu5 in Urothelial Carcinoma. PLoS ONE.

[25] Wang W., Liu F., Wang C., Wang C., Tang Y., Jiang Z. (2017). Glutathione S-transferase A1 mediates nicotine-induced lung cancer cell metastasis by promoting epithelial-mesenchymal transition. Exp. Ther Med..

[26] Huang Z.Z., Chen C., Zeng Z., Yang H., Oh J., Chen L., Lu S.C. (2001). Mechanism and significance of increased glutathione level in human hepatocellular carcinoma and liver regeneration. FASEB J..

[27] Carretero J., Obrador E., Anasagasti M.J., Martin J.J., Vidal-Vanaclocha F., Estrela J.M. (1999). Growth-associated changes in glutathione content correlate with liver metastatic activity of B16 melanoma cells. Clin. Exp. Metastasis.

[28] Traverso N., Ricciarelli R., Nitti M., Marengo B., Furfaro A.L., Pronzato M.A., Marinari U.M., Domenicotti C. (2013). Role of glutathione in cancer progression and chemoresistance. Oxid. Med. Cell Longev..

[29] Zhang H.X., Chen Y., Xu R., He Q.Y. (2018). Nrf2 mediates the resistance of human A549 and HepG2 cancer cells to boningmycin, a new antitumor antibiotic, in vitro through regulation of glutathione levels. Acta Pharm. Sin..

[30] Hayes J.D., Flanagan J.U., Jowsey I.R. (2005). Glutathione transferases. Annu. Rev. Pharmacol. Toxicol..

[31] Harbottle A., Daly A.K., Atherton K., Campbell F.C. (2001). Role of glutathione S-transferase P1, P-glycoprotein and multidrug resistance-associated protein 1 in acquired doxorubicin resistance. Int J. Cancer.

[32] Sawers L., Ferguson M.J., Ihrig B.R., Young H.C., Chakravarty P., Wolf C.R., Smith G. (2014). Glutathione S-transferase P1 (GSTP1) directly influences platinum drug chemosensitivity in ovarian tumour cell lines. Br. J. Cancer.

[33] Li J., Wang Q., Yang Y., Lei C., Yang F., Liang L., Chen C., Xia J., Wang K., Tang N. (2019). GSTZ1 deficiency promotes hepatocellular carcinoma proliferation via activation of the KEAP1/NRF2 pathway. J. Exp. Clin. Cancer Res..

[34] Sun C., Gu Y., Chen G., Du Y. (2019). Bioinformatics Analysis of Stromal Molecular Signatures Associated with Breast and Prostate Cancer. J. Comput. Biol..

[35] Wang Y., Lei L., Chi Y.G., Liu L.B., Yang B.P. (2019). A comprehensive understanding of ovarian carcinoma survival prognosis by novel biomarkers. Eur. Rev. Med. Pharm. Sci..

[36] Hentschel A.E., Nieuwenhuijzen J.A., Bosschieter J., Splunter A.P.V., Lissenberg-Witte B.I., Voorn J.P.V., Segerink L.I., Moorselaar R., Steenbergen R.D.M. (2020). Comparative Analysis of Urine Fractions for Optimal Bladder Cancer Detection Using DNA Methylation Markers. Cancers.

[37] Hoque M.O., Begum S., Topaloglu O., Chatterjee A., Rosenbaum E., Van Criekinge W., Westra W.H., Schoenberg M., Zahurak M., Goodman S.N. (2006). Quantitation of promoter methylation of multiple genes in urine DNA and bladder cancer detection. J. Natl. Cancer Inst..

[38] Urakami S., Shiina H., Enokida H., Kawakami T., Kawamoto K., Hirata H., Tanaka Y., Kikuno N., Nakagawa M., Igawa M. (2006). Combination analysis of hypermethylated Wnt-antagonist family genes as a novel epigenetic biomarker panel for bladder cancer detection. Clin. Cancer Res..

